# Laparoscopic Salpingectomy Cancellations: Patient-Reported Reasons and Areas for Improvement

**DOI:** 10.7759/cureus.102461

**Published:** 2026-01-28

**Authors:** Megan Masten, Sofie Rosenberg, Claire Schultz, Nicole A Larrea

**Affiliations:** 1 Obstetrics and Gynecology, MaineHealth, Portland, USA; 2 Obstetrics and Gynecology, University of Colorado School of Medicine, Aurora, USA; 3 Obstetrics and Gynecology, University of Vermont Health, Burlington, USA; 4 Obstetrics and Gynecology, Denver Health and Hospitals, Denver, USA

**Keywords:** bilateral salpingectomy, cancellation, permanent contraception, sterilization, tubal ligation

## Abstract

Background and objective

Although permanent surgical contraception via laparoscopic salpingectomy is commonly performed and associated with a substantial cancellation rate, the reasons for cancellation are poorly characterized. This study sought to examine patient-reported reasons for sterilization cancellation.

Materials and methods

Patients aged 18 to 50 who cancelled their laparoscopic salpingectomy at our academic county hospital and who were English- or Spanish-speaking were contacted for a qualitative interview. Inductive coding was used.

Results

Fifteen phone interviews were conducted between January 2023 and February 2024. Nine of the 15 participants (60%) were confident in their desire for permanent contraception, and eight of 15 (53.3%) continued to desire laparoscopic salpingectomy. The most common factor contributing to surgery cancellation among participants who desired permanent contraception was financial concerns, with insurance issues cited most frequently.

Conclusions

Some patients change their minds about undergoing salpingectomy. Financial concerns remain a barrier to receiving laparoscopic salpingectomy for permanent contraception, even with full insurance coverage or deeply discounted services.

## Introduction

Permanent contraception via tubal sterilization is the preferred contraceptive method for approximately 25% of women in the United States [1,2]. However, the methods and timing of tubal sterilization differ. Tubal sterilization procedures can be performed in the immediate postpartum period or laparoscopically via salpingectomy at a time remote from pregnancy, which is termed interval tubal sterilization or laparoscopic permanent contraception [3]. There are many known barriers to immediate postpartum permanent contraception, including failure to sign the appropriate Medicaid form in advance, maternal medical conditions, and limited operating room (OR) space [4-13]. Barriers to interval laparoscopic permanent contraception are less well understood, although data reveal a high rate of surgical cancellation [14,15].

At a suburban New York City safety-net hospital, laparoscopic salpingectomy cancellation was reported to be 28%, and at our academic tertiary care hospital and academic county hospital in Denver, Colorado, the combined cancellation rate of permanent contraception via laparoscopic sterilization was 22% [14,15]. Our previous retrospective investigation using chart review of cancellation rates revealed that 74% of individuals cancelled their surgery for patient-related reasons, such as surgery no longer desired or scheduling conflict, followed by cancellation due to financial concerns, with only 22% ultimately completing surgery within one year [15]. The majority of patients cancelled within less than a week of their scheduled surgery date (71.5%), with approximately one-third (32.5%) cancelling on the same day, either calling or not showing on the day of their surgery [15]. Short-notice cancellation of surgical procedures results in decreased OR utilization, which decreases others’ access to needed surgical care and raises concerns regarding barriers to surgical completion.

Currently, there are no published studies that prospectively examine cancellations of laparoscopic salpingectomy for permanent contraception to determine patient-reported reasons for cancellation. Understanding why these surgeries are cancelled may enable clinicians and hospital systems to design targeted interventions to improve access to laparoscopic salpingectomy when it is a patient’s ultimate contraceptive method of choice and to better support patients through their decision-making process when they desire another contraceptive method. This study aims to explore patient-reported qualitative reasons for cancellation of laparoscopic salpingectomy, to inform future intervention development. This study has limitations, as the authors used a chart review to analyze patients’ reasons for cancellation of laparoscopic salpingectomy for permanent contraception [15]. Chart review is not the ideal method for exploring patient-related cancellation, given the lack of consistent and clear documentation in the electronic health record.

## Materials and methods

Study participation was offered to patients aged 18-50 years who scheduled and subsequently cancelled their laparoscopic salpingectomy for permanent contraception at our academic-affiliated, safety-net, county hospital in Denver, Colorado. Participants were English or Spanish-speaking. Given that this was mainly an exploratory study, a sample size was not pre-determined. A semi-structured phone interview was conducted with all patients who met the inclusion criteria and included the following questions (Table 1).

**Table 1:** Telephone survey questions

Questions
1) It looks like you scheduled a laparoscopic salpingectomy, also known as a tubal ligation or a surgery, to tie your tubes at [our hospital] for (date). Is that correct?
2) After looking at your chart, it looks like you did not have the surgery done. There can be many reasons why people do not go forward with the surgery. What contributed to your decision to not go forward with this surgery?
3) Did you encounter any problems that contributed to cancelling your surgery? These could be problems that occurred in your own life or problems in interacting with the clinic and hospital. If yes, what problems did you face? What are your plans for birth control or contraception now that you have cancelled your surgery?
4) (For those who state they would still like a salpingectomy): What could [our hospital] have done to help make it easier for you to complete your surgery?
5) Is there anything else you’d like to discuss about your surgery that we haven’t yet talked about?

After completion of the telephone survey, an online survey was sent to participants’ preferred email addresses through REDCap [16,17]. REDCap survey questions were multiple choice and focused on reasons for scheduling surgery, access issues, and participants’ perceptions of counseling and confidence. Participants were given gift cards after completing both the telephone and online surveys. A qualitative review of responses was performed using inductive coding. Two interviewers (MM and SR) conducted the interviews and coded the responses independently. Codes were reviewed and revised for consensus, and all conflicts were resolved through agreement.

## Results

Sixty-three patients who met the inclusion criteria were contacted between January 2023 and February 2024. Fifteen telephone interviews were completed. Patient demographics are listed in Table 2.

**Table 2:** Phone interview participant demographics (N=15)

Variables	Values
Age, years, median (range)	32 (25-40)
Gravidity, median (range)	3 (0-9)
Parity, median (range)	2 (0-7)
Ethnicity - Hispanic/Latinx, n (%)	9/15 (60%)
Ethnicity - non-Hispanic/Latinx, n (%)	6/15 (40%)
Race - White/Caucasian, n (%)	10/15 (66.7%)
Race - other, n (%)	3/15 (20%)
Race - mixed, n (%)	1/15 (6.7%)
Race - unknown, n (%)	1/15 (6.7%)
Insurance – public, n (%)	60% (9/15)
Insurance – private, n (%)	26.7% (4/15)
Insurance - emergency, n (%)	13.3% (2/15)

Nine out of fifteen participants (60%) were confident about desiring permanent contraception and still desired laparoscopic salpingectomy (8/15, 53.3%). Participants strongly desiring permanent contraception expressed financial concerns, expressing sentiments such as:

“And my husband and I, we were completely ready to do this surgery, it's not that we changed our minds, it wasn’t that, it was because we couldn’t afford it.”

Another participant stated:

“It’s a little bit… frustrating because I really, really still want it, but the problem is now, I have to do… the research of finding additional ways to pay for it.”

A minority (2/15, 13.3%) of participants felt sure they did not want laparoscopic salpingectomy, and an additional 4/15 (26.7%) were unsure due to potential desire for future fertility, changes in relationship status, and other issues. For example:

"And then there was… a lingering of a very minute chance that I might change my mind about pregnancy in the future, and thinking... ‘this is super final, and I can't always predict how my moods will change’ although I am pretty certain that I don't want kids now, but that might change down the road.”

The most common factor contributing to surgery cancellation for participants who desired permanent contraception was financial concerns, with insurance issues being most frequently cited within this category (Table 3). Four out of the six individuals who reported financial concerns also cited high copays as a barrier. Additionally, private insurance prevented some participants from receiving care at our institution due to their specific insurance plans (13.3%, 2/15). Other barriers included a lack of childcare and transportation issues. Of note, a single participant interview may include multiple sentiments within the same theme, and the reported frequency reflects this.

**Table 3:** Reasons for cancellation among participants

Reason for cancellation	Frequency cited
Barriers to care	22
Childcare	7
Health concerns	3
Life issues	6
Emergency	2
Partner separation	2
Medicaid sterilization consent form/paperwork	1
Negative research study experience	2
Not having a concrete time for the operating room (OR)	1
Open to other contraceptive options	2
Partner desires more children	1
Payment issue/financial concern	21
Unable to work	1
Insurance coverage	10
Require discount	3
Recovery time	4
Transportation	1
Wants to avoid surgery	1
Unsure about surgical permanent contraception	7
Does not want surgical permanent contraception	3
Political concerns	1

Many participants reported a positive experience with the hospital and provided suggestions to reduce barriers to surgery, including receiving a confirmed OR time, modifying Medicaid sterilization consent form requirements, and reducing costs or improving cost transparency for the procedure. Seven participants partially or fully completed surveys. Although there were limited survey responses, all participants agreed that their doctor counseled them thoroughly (5/7, 71.4% strongly agree, 2/7, 28.5% agree) and that they knew everything they needed to know before surgery (4/6, 66.7% strongly agree, 2/6, 33.3% agree).

Our team conducted an additional analysis of the financial impact of laparoscopic salpingectomy cancellations. OR time was reassigned, and minimal time was lost for eight of 15 scheduled cases (53.3%). However, a total of 13 OR hours were still lost due to canceled cases, resulting in losses exceeding $30,000 based on facility and provider costs.

## Discussion

Our study characterizes patient-reported reasons for high cancellation rates of laparoscopic salpingectomy. Some patients ultimately decide not to pursue permanent contraception; in this study, 40% of participants did not want to pursue permanent contraception. In these cases, the preferred outcome is that patients do not undergo surgery if it does not align with their goals. Ideally, this decision would be made before surgery is scheduled, though this is not always feasible. These participants frequently emphasized the high quality of care they received and reported feeling well-informed about the procedure, but changed their minds after scheduling laparoscopic salpingectomy. For this subset of patients, cancellations occurring after scheduling may be unavoidable. Notably, the majority of participants in this study continued to desire laparoscopic salpingectomy as a method of permanent contraception even after their surgery was canceled (53.3%). Although financial concerns were the most commonly cited reason for cancellation, this finding is inconsistent with actual financial coverage, as most insurance types, including discounted services, fully cover the procedure. This discrepancy suggests that a breakdown in communication between the hospital and patients regarding financial coverage may be a contributing factor.

Cancellation rates were not calculated in this study. Previous research has documented high cancellation rates for laparoscopic salpingectomy performed for permanent contraception [14,15]. Prior studies also report that cancellations commonly occur on short notice, often less than a week before the scheduled surgery date [15]. Such cancellations can result in wasted resources, time, and effort for the healthcare system, and importantly, may reflect underlying issues with accessibility [18].

Strengths of this study include the fact that it represents the first published collection of patient-reported reasons for cancellation of laparoscopic salpingectomy for permanent contraception. Because this procedure has a high cancellation rate, which can lead to wasted resources and highlight accessibility challenges, understanding the underlying reasons for cancellation could inform policy and clinical practices aimed at reducing this problem. 

Limitations of this study include its small sample size and the fact that it involved a single academic-affiliated, safety-net, county hospital, which may limit generalizability to other settings. Patients receiving care at different institutions may not experience the same barriers, particularly regarding financial concerns. Participant recruitment was challenging, with fewer than 25% of eligible patients successfully contacted by phone and agreeing to participate. Because only a small proportion of patients meeting the inclusion criteria participated, selection bias may have influenced the responses, and important barriers could have been missed. Finally, as this study relied on retrospective, patient-reported reasons for laparoscopic salpingectomy cancellation, recall bias may have affected the results.

Future studies could focus on system-level interventions, including enhanced transparency and structured discussions among financial counselors, surgery schedulers, and patients regarding the cost and insurance coverage of this procedure. In our patient population, improving transparency and providing discussions with financial counseling would likely be the most feasible and impactful intervention. Other quality improvement initiatives could include recognizing that some patients may change their minds about laparoscopic salpingectomy during counseling or the preoperative visit, and encouraging them to inform the surgical team as early as possible, ideally more than a week before the scheduled surgery. Additionally, surgery schedulers could implement proactive measures, such as contacting patients approximately two weeks before the procedure to confirm their intent to proceed, allowing earlier cancellations if preferences have changed, or connecting patients with financial counseling if cost concerns remain.

Barriers to care, including cost, are impacting individuals’ ability to obtain their preferred method when that option is a laparoscopic salpingectomy. This forces individuals to choose other contraceptive options, thereby reducing patient autonomy and reproductive choice. Ultimately, addressing these barriers is essential to promoting equitable, patient-centered care in which individuals can access their chosen contraceptive method.

## Conclusions

Laparoscopic salpingectomy is a commonly desired, safe, and effective method of permanent contraception. Our study indicates that most patients who cancel their surgery continue to desire laparoscopic salpingectomy, and that financial concerns are a frequent contributor to cancellations. Canceled procedures result in financial losses for the hospital, wasted OR time, and patients not receiving the care they want and need. Some patients may change their minds about undergoing laparoscopic salpingectomy, sometimes after scheduling, and these cancellations may be unavoidable. Based on interviews with patients who canceled, potential interventions include increased financial transparency, offering payment plans, and providing more concrete OR times to facilitate childcare and transportation planning. Most importantly, addressing cost as a barrier to care could improve access to laparoscopic salpingectomy.
